# Draft genome sequence of *Listeria innocua* AZLIn05 isolated from beef seekh kebab in Bangladesh

**DOI:** 10.1128/mra.00263-26

**Published:** 2026-05-18

**Authors:** Ashrafus Safa, Md. Imran Khan Masum, Zobayda Nahar, Farishta Shahel, Raidah Jahan, Margia Hossain Rahi, Hamja Hasanat, Jinath Sultana Jime, Nayeema Bulbul, Md. Fakruddin

**Affiliations:** 1 Department of Life Sciences, School of Environment and Life Sciences, Independent University, Bangladesh (IUB) 117116, Dhaka, Bangladesh; 2 Department of Biochemistry and Microbiology, School of Health and Life Sciences, North South University 54495, Dhaka, Bangladesh; Nanchang University, Nanchang, Jiangxi, China

**Keywords:** *Listeria innocua*, genome, food-borne pathogens

## Abstract

This report presents the draft genome sequence of *Listeria innocua* strain AZLIn05, isolated from seekh kebab—a meat-based South Asian cuisine—in Dhaka, Bangladesh. The genome had a length of 2,832,024 bp, a G + C content of 37.4%, and 2,813 coding DNA sequences, 17 tRNAs, 1 tmRNA, and 2 rRNA genes.

## ANNOUNCEMENT

*Listeria innocua* is generally a nonpathogenic species but has been linked to meningitis in immunocompromised individuals (1, 2). Until now, it has been isolated only from raw chicken and cattle farms in Bangladesh. This study reports the draft genome of strain AZLIn05 from a ready-to-eat beef seekh kebab—for the first time in Bangladesh.

The 25 g sample was collected from a street vendor in Dhaka, Bangladesh (23.7664°N, 90.3587°E); enriched in sterilized Buffered *Listeria* Enrichment Broth (Liofilchem, Italy) at 30°C for 48 h, subsequently plated onto Oxford Agar (Liofilchem, Italy) and incubated aerobically at 35°C for 48 h. The suspected *L. innocua* was isolated in Tryptic Soy Agar (Liofilchem, Italy) with yeast extract and identified using *L. innocua*-specific primers targeting the *lin0406* gene (forward primer: 5′-CGCATTTATCGCCAAAACTC-3′; reverse primer: 5′-TCGTGACATAGACGCGATTG-3′) (3). Genomic DNA was extracted from a pure colony grown overnight in LB broth using the Qiagen Mini Kit (Qiagen, Germany) according to the manufacturer’s protocol. DNA purity and concentration were assessed using a NanoDrop (Thermo Fisher Scientific, USA). The library preparation was performed using the Nextera XT Library Preparation Kit (Illumina, USA). The libraries were sequenced using the Illumina HiSeq 2500 with paired-end read sizes of 150 bp, and the genome coverage was 46×. For *de novo* assembly, 8.2 M paired-end reads for strain AZLIn05 were used. The raw sequence data were quality-checked with FastQC v0.12.0 (4),and trimmed with Trimmomatic v0.39 (5). To assemble the short reads, we used Unicycler v0.4.9 (6). Genome completeness was assessed using CheckM v1.2.4 (7). Prokka v1.14.6 (8) was used for rapid prokaryotic genome annotation. Acquired antibiotic resistance genes and virulence factors were identified using ABRicate v1.0.1 (9). The pathogenic potential of the AZLIn05 strain was assessed using the PathogenFinder-1.1 server (10). Additionally, the strain’s multilocus sequence typing (MLST) profile was determined by comparing the assembled sequences against the *Listeria* sequence typing database on BIGSdb using BLAST (11). Default parameters were used for all software unless otherwise specified.

The draft genome of AZLIn05 is 2,832,024 bp and has a G + C content of 37.4%. The genome was distributed into 40 contigs with an *N*_50_ of 230,971. CheckM v1.2.4 recognized that the genome was 99.31% complete and 0.06% contaminated. Genome analysis revealed 2,813 coding sequences, 17 tRNAs, two rRNAs, and one tmRNA. The presence of two rRNA genes in the AZLIn05 genome, which is fewer than six rRNA operons in *L. innocua* CLIP11262 (Ref), is likely due to the repetitive nature of rRNA loci and their underrepresentation in short-read draft assemblies. Comparative analysis of the 16S rRNA gene using BLASTn showed 100% sequence identity and 97% query coverage with *L. innocua* strain ATCC 33090. Antibiotic-resistant genes for lincosamide and fosfomycin antibiotics were detected, along with 13 virulence-associated genes. Although *L. innocua* is typically considered nonpathogenic, strain AZLIn05 was predicted to be a human pathogen using the PathogenFinder-1.1 server. Furthermore, the allelic profile determined for the seven housekeeping genes—*abcZ*, 188; *bglA*, 157; *cat*, 182; *dapE*, 223; *dat*, 136; *ldh*, 353; and *lhkA*, 148—classified AZLIn05 into the sequence type ST-1085.

## Data Availability

The *L. innocua* strain AZLIn05 whole-genome shotgun project has been deposited in DDBJ/ENA/GenBank under accession number JBKYKH000000000.1. The raw reads were deposited in the NCBI SRA database with accession number SRR31937442. The NCBI reference sequence accession numbers used for *L. innocua* were NC_003212.1 and NR_116805.1.
